# Colonic Ganglioneuroma—A Rare Finding During Colonoscopy

**DOI:** 10.3390/diagnostics15212716

**Published:** 2025-10-27

**Authors:** Anita Sejben, Tamás Lantos

**Affiliations:** 1 Department of Pathology, University of Szeged, 6725 Szeged, Hungary; 2 Department of Medical Physics and Informatics, University of Szeged, 6720 Szeged, Hungary

**Keywords:** colon polyp, ganglioneuroma, PTEN-hamartoma tumour syndrome

## Abstract

A 66-year-old female underwent a colonoscopy for persistent left lower abdominal discomfort and mucous stool passage. Endoscopic examination revealed a 3 mm sessile polyp in the ascending colon. Histopathological examination of the polyp showed preserved crypt architecture with a monomorphic spindle-cell proliferation within the lamina propria. Scattered ganglion cells were present and demonstrated immunoreactivity for Calretinin and S100. The lesion was diagnosed as a colonic ganglioneuroma. Ganglioneuromas are rarely localised to the gastrointestinal tract and often detected incidentally during colonoscopic screening. While solitary lesions are typically sporadic; diffuse or multiple ganglioneuromas may be associated with hereditary syndromes such as neurofibromatosis type 1, multiple endocrine neoplasia type 2B, and juvenile polyposis, the latter belonging to the PTEN-hamartoma tumour syndrome spectrum. Clinically, most colonic ganglioneuromas are asymptomatic, although some may present with nonspecific gastrointestinal symptoms. Despite their benign nature, rare cases of malignant transformation and association with adenocarcinoma have been reported.

**Figure 1:**
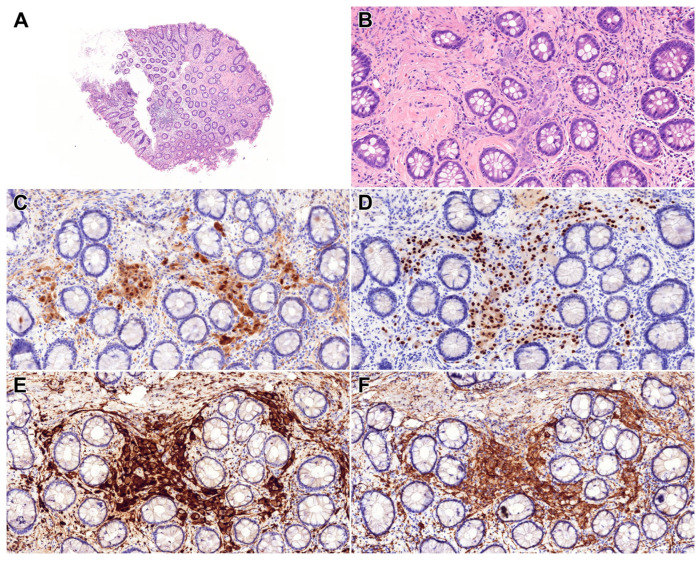
A 66-year-old female patient with a medical history significant for benign essential hypertension, follicular nodular disease of the thyroid, and arthrosis was referred to colonoscopy due to persistent left lower abdominal discomfort lasting for 2 weeks and passage of a mucous stool. During colonoscopic evaluation, a 3 mm polyp (Paris Is, Kudo IIIs) was identified in the ascending colon and successfully removed using a cold snare technique. Examination of the sigmoid colon revealed multiple diverticula, situated within a mildly hyperemic mucosal environment. Microscopic evaluation of the resected polyp demonstrated preserved crypt architecture. Within the lamina propria between intact crypts, a monomorphic spindle-cell proliferation was observed ((**A**,**B**), HE, 5× and 20×). Preserved ganglion cells were identified and exhibited immunoreactivity for Calretinin ((**C**), Calretinin, 20×). The spindle-cell component also demonstrated positivity for PHOX2B ((**D**), PHOX2B, 20×), S100 ((**E**), S100, 20×), and Synaptophysin ((**F**), Synaptophysin, 20×), indicating neural differentiation [1]. Immunohistochemical staining for EMA and GLUT1 was negative. The polyp was completely excised, and based on morphological and immunohistochemical findings, the lesion was diagnosed as a colonic ganglioneuroma. Ganglioneuromas are benign tumours of mesenchymal origin that predominantly arise in the head and neck region or the adrenal glands; gastrointestinal involvement is uncommon, and epidemiological data remain limited [2,3,4]. While most cases are sporadic and are referred to as polypoid ganglioneuromas, based on the description of the current WHO classification, 40% of patients with ganglioneuromas have diffuse ganglioneuromatosis, which may occur in association with hereditary syndromes, including neurofibromatosis type 1, multiple endocrine neoplasia (MEN) type 2B, and juvenile polyposis [2,5,6,7,8]. Solitary colonic ganglioneuromas have also been reported in patients with Cowden syndrome (which forms part of the PTEN-hamartoma tumour syndrome spectrum), tuberous sclerosis, polyposis coli, and juvenile polyposis, as well [2,5,9,10]. Clinical manifestations include solitary ganglioneuroma, diffuse ganglioneuromatosis, and ganglioneuromatous polyposis [2]. Gastrointestinal ganglioneuromas are most frequently localised to the left colon and rectum. Clinically, they are often asymptomatic and may be incidentally detected during routine surveillance colonoscopies [5]. However, when symptomatic, they may present with constipation or diarrhoea, abdominal distension, intussusception, megacolon, or hematochezia [2,4,11,12,13,14]. Endoscopically, ganglioneuromas appear as uncharacteristic mucosal polyps generally not exceeding 2 cm in diameter. Histologically, they are composed of mature ganglion cells and satellite cells may be accompanied by eosinophilic granulocyte infiltration [2,11]. Although ganglioneuromas are benign, rare cases of malignant transformation have been reported, including associations with adenocarcinoma [15]. Currently, endoscopic treatment is considered sufficient, especially in solitary cases [16]. Given their potential association with hereditary syndromes, the identification of such tumours should prompt a thorough evaluation for accompanying clinical features suggestive of systemic involvement.

## Data Availability

The original contributions presented in this study are included in the article. Further inquiries can be directed to the corresponding author.

## Abbreviations

The following abbreviations are used in this manuscript:

EMA	Epithelial membrane antigen
GLUT1	Glucose transporter 1
HE	Hematoxylin and eosin
MEN	Multiple endocrine neoplasia
PHOX2B	Paired-like homeobox 2B
PTEN	Phosphatase and tensin homolog
S100	S100 protein
WHO	World Health Organization
